# Smartphones and Mental Health Awareness and Utilization in a Low-Income Urban Community: Focus Group Study

**DOI:** 10.2196/65650

**Published:** 2025-11-20

**Authors:** Nadia Alam, Domenico Giacco, Bulbul Siddiqi, Swaran Singh, Sagar Jilka

**Affiliations:** 1 Warwick Medical School, University of Warwick, Gibbet Hill Road, Coventry, CV4 7AL, United Kingdom, 44 07300311294; 2 Warwick Centre for Global Health, University of Warwick, Coventry, United Kingdom; 3 Coventry and Warwickshire Partnership NHS Trust, Coventry, United Kingdom; 4 Department of Political Science and Sociology, North South University, Dhaka, Bangladesh; 5 Institute of Psychiatry, Psychology and Neuroscience, King's College London, London, United Kingdom

**Keywords:** mental health, mHealth, lower- and middle-income countries, smartphones, awareness, developing countries, focus group study, mental health disorders, LMICs, digital tools, digital health, digital mental health, thematic analysis

## Abstract

**Background:**

Mental health disorders pose a significant challenge in low- and middle-income countries (LMICs), contributing substantially to the global disease burden. Despite the high prevalence of these disorders, LMICs allocate less than 1% of health budgets to mental health, resulting in inadequate care and a severe shortage of professionals. Stigma and cultural misconceptions further hinder access to mental health services. These challenges are present in Bangladesh, with high prevalence rates of depression and anxiety, as well as a centralized and underresourced mental health care system. Digital tools, such as smartphone apps and online platforms, offer innovative solutions to these challenges by increasing accessibility, cost-effectiveness, and scalability of mental health interventions.

**Objective:**

This study aims to characterize the views around digital tools for mental health among residents of Korail (a major slum in Dhaka, Bangladesh), including the use of smartphones, and investigate acceptable digital tools and barriers and facilitators for digital mental health tools.

**Methods:**

A total of 8 focus group discussions were conducted with 38 participants, including individuals with serious mental disorders and their caregivers. The focus group discussions were guided using a semistructured topic guide, which included broad questions on smartphone usage to contextualize digital access, primarily focusing on perceptions of using mobile technology for mental health care. Focus groups were held in Bangla, audio recorded, and transcribed and translated in English. Data were analyzed using thematic analysis in NVivo 14.

**Results:**

Participants (mean age 37 y, SD 13.7) were mostly female (30/38, 79%), and 45% (17/38) personally owned smartphones, although 92% (35/38) reported smartphone access within the household. The findings revealed a general lack of awareness and understanding of digital mental health tools among slum residents. However, there was a notable appetite for these tools; participants recognized their potential to provide timely and cost-effective support, reduce hospital visits, and make health care more accessible. Participants highlighted the convenience and communication benefits of smartphones but expressed concerns about misuse such as excessive use, particularly among adolescents. Barriers to the utilization of digital mental health tools included limited technological literacy and accessibility issues. Despite these challenges, participants acknowledged the potential of these tools to bridge the gap in mental health services, especially for those unable to travel. The importance of providing proper guidance and education to maximize the effectiveness of digital tools was emphasized.

**Conclusions:**

Digital mental health tools hold promise for improving mental health care in underserved slum communities. This study underscores the need for further research and investment in tailored digital mental health solutions to address the unique needs of slum populations in LMICs.

## Introduction

### Mental Health in Low- and Middle-Income Countries

Mental health disorders significantly contribute to the global burden of disease, with 11.1% of the total burden in low- and middle-income countries (LMICs) attributed to mental health issues [1-4]. The World Health Organization reports that unipolar depressive disorder, schizophrenia, and bipolar disorder are among the leading causes of disability in these regions [5]. Furthermore, substance use disorders, particularly alcohol use, account for a significant portion of the disease burden in LMICs [6]. Despite the high prevalence of mental health disorders, resources are severely limited [2]. Less than 1% of health budgets in many LMICs are allocated to mental health, resulting in inadequate care for the majority of those affected [7].

### Mental Health in Bangladesh and Slums

Mental health disorders are prevalent in Bangladesh, affecting approximately 16.1% of the population [8], yet they are often underreported and inadequately addressed due to limited resources, infrastructure, and a severe shortage of mental health professionals, with only 0.5 psychiatrists and 0.2 psychologists per 100,000 people [9]. In urban slum areas like Korail in Dhaka, mental health disorders are disproportionately high due to adverse living conditions such as poverty, overcrowding, and lack of basic services, with prevalence rates 30%-50% higher than in non-slum urban areas [10-15]. These environments are marked by high levels of violence, substance abuse, and chronic stress, exacerbating mental health issues while social support networks are often weak or absent, leading many to rely on informal support or go without care [16]. Efforts to improve mental health services, including policy initiatives and the establishment of mental health units in district hospitals, have been slow, with services often underfunded and lacking trained professionals [2,8,17]. This highlights the urgent need for innovative solutions to address mental health care in these regions.

### Mental Health and Digital Tools

Digital tools, such as smartphone apps and online platforms, have been utilized to deliver mental health interventions such as cognitive-behavioral therapy and mindfulness training [18], provide psychoeducation, and offer support networks [19-21]. These tools can bridge the gap in mental health services, especially in regions with limited access to traditional mental health care [22]. Digital tools offer several advantages, including accessibility, cost-effectiveness, and scalability [23]. Furthermore, digital platforms can facilitate self-monitoring and provide users with real-time feedback and support, enhancing the effectiveness of mental health interventions [24].

Evidence demonstrates that some LMICs have successfully adopted digital technology to raise awareness, screen, diagnose, and manage common mental health disorders for wider populations [23,25-28]. For instance, in India, mobile health (mHealth) initiatives have been used to deliver mental health services to rural and underserved populations, showing promising results in improving access and outcomes [28]. Similarly, in Kenya, digital platforms have been utilized to provide mental health education and support, significantly enhancing mental health literacy and reducing stigma [29]. A study in South Africa found that a mobile phone–based intervention for depression was effective in reducing symptoms and improving quality of life among participants [30]. Another example is the use of telepsychiatry in remote areas of Brazil, which has improved access to mental health care and reduced the need for travel to urban centers [31]. These examples highlight the potential of digital tools to address the mental health care gap in LMICs.

Digital tools can be particularly useful in impoverished communities like slums, where traditional mental health services are scarce [2,11,14], but smartphone ownership is vast [32]. However, understanding slum residents’ perception of smartphones and digital tools for mental health is crucial for the successful implementation of these technologies. Perceptions influence the acceptance and utilization of digital interventions, and addressing concerns such as privacy, usability, and cultural relevance is essential [33]. Understanding general patterns of smartphone use in this community provides critical context for interpreting how residents might engage with or resist mobile mental health solutions. Engaging communities in the design and implementation process can enhance the effectiveness and sustainability of digital mental health programs [34].

Given the increasing availability of smartphones in low-resource settings and the growing potential of digital tools to support mental health care, it is essential to understand how these tools are perceived by underserved populations. This study therefore aimed to explore the awareness, perceptions, and acceptance of smartphone-based mental health tools among residents of Korail slum in Dhaka, Bangladesh. Specifically, the objectives were to assess familiarity with digital mental health tools, identify barriers and facilitators to their use, and understand how such tools might support mental health care in this context.

## Methods

### Study Design

This research formed part of a larger collaborative research project on mental health in slums of LMICs [11]. This study employed a qualitative research design to explore perceptions on smartphones and use of digital tools for mental health among residents of the Korail slum in Dhaka, Bangladesh. The qualitative approach was chosen to gather in-depth insights into the participants’ perceptions and attitudes toward digital tools for mental health. The study is part of a larger mixed-methods project aimed at understanding and predicting mental health relapse using digital phenotyping in LMICs.

### Study Site and Participants

This study was conducted in Korail slum, Dhaka, Bangladesh, through group discussions with patients and caregivers. Korail is one of the largest slums in Bangladesh, located in the center of the city, encroaching on parts of Dhaka’s most affluent neighborhoods. The majority of Korail’s population (estimated to be around 200,000) [12] subsist below the poverty line and work in low-income jobs. They have limited access to the city’s health care services, and there is little connection with the surrounding major roads [35]. Participants had to have been residents of the Korail slum, aged 18 years or older, and either diagnosed with a serious mental disorder (SMD) or caregivers of individuals diagnosed with SMD. We used purposive sampling to ensure inclusion of individuals with lived experience of SMDs and their caregivers, reflecting diverse perspectives on smartphone and digital health tool usage. Recruitment was facilitated by community engagement representatives associated with the TRANSFORM (Transforming Access to Care for Serious Mental Disorders in Slums) Project [11], who are trusted members of the local community. These trusted contacts played a key role in introducing the study to potential participants and delivering culturally sensitive explanations about its purpose and procedures. Recruitment was conducted through informal community engagement strategies, including conversations at local tea stalls and the TRANSFORM project field office. No public advertisements were used. All potential participants received an information sheet and were given at least 24 hours to consider participation.

### Ethical Considerations

#### Human Subject Ethics Review Approvals

This study received ethical clearance from the Biomedical and Scientific Research Ethics Committee at the University of Warwick (reference number: BSREC 100/22‐23). It formed part of the wider National Institute for Health and Care Research–funded TRANSFORM project [11], which underwent rigorous ethical review both in the United Kingdom and by partner institutions in Bangladesh to ensure compliance with local ethical standards.

A safeguarding and risk management plan was developed to prioritize participant well-being throughout the study. Given the sensitive nature of the topic—discussions about personal or family experiences with mental illness—there was potential for emotional distress. Data collection was led by NA—a trained researcher and doctoral candidate with a background in psychology who was trained in research ethics and working with vulnerable populations. The safeguarding protocol allowed for immediate responses if a participant showed signs of distress, including pausing or stopping the session, checking in with the participant, and offering referral to psychiatric support available through the local study team.

#### Informed Consent

Informed written consent was obtained from all participants before data collection activities began. Study information sheets and consent forms were provided in both English and Bengali to ensure accessibility. Participants were given a minimum of 24 hours to consider their involvement. For participants with lived experience of mental illness, we used a previously validated capacity assessment tool from the TRANSFORM project to confirm ability to consent. In cases where participants could not sign, a thumbprint was collected in the presence of a witness. Verbal consent was also recorded at the beginning of each focus group. Participants were informed of their right to withdraw at any time without providing a reason.

#### Privacy and Confidentiality

All participant data were anonymized and stored on encrypted devices in compliance with the UK General Data Protection Regulation and the UK Data Protection Act (2018). Signed consent forms were stored separately in locked filing cabinets. Focus group discussion (FGD) transcripts were deidentified before analysis, and no personally identifying information is included in this paper or any outputs. Digital files were stored on secure, access-restricted university servers.

#### Compensation

Each participant received compensation of 800 Bangladeshi Taka (approximately £5.30/US $6.60) to cover their time and any expenses. This amount reflected local wage and travel standards, especially considering that Korail is a large urban slum where travel to field offices requires time and costs. They were assured that compensation was not conditional upon completion of the study and would still be given in the event of early withdrawal.

#### Participant Identification in Study Materials

No identifying information will be disclosed in any study publications or supplementary materials. All data were anonymized prior to analysis, and each participant was assigned a unique code to maintain confidentiality.

### Data Collection

The FGDs were conducted in the Korail slum, within the established research office of the TRANSFORM Project [11]. The FGDs were structured to ensure a diverse representation of the community, including different genders, ages, and socioeconomic status. Each session was moderated by a trained facilitator, with the assistance of a notetaker and qualitative researcher, and lasted for a duration of 30‐45 minutes. The moderator (NA) guided the discussions using a semistructured topic guide, which included broad questions on smartphone usage to contextualize digital access, but the primary emphasis was on discussing perceptions of using mobile technology for mental health care, including reactions to examples of existing platforms. As part of the discussion, participants were introduced to examples of existing Bangladeshi digital health tools used on smartphones, including Doctime (a telemedicine platform) [36] and Maya Apa (a mobile app offering health and mental well-being support) [37], to help them conceptualize how digital mental health support might be delivered. The FGDs were audio recorded in Bangla, the local language, to ensure accurate capture of the discussions. The recordings were then transcribed verbatim and translated into English. To maintain confidentiality, all personal identifiers were removed during transcription. The translated transcripts were reviewed by the research team to ensure accuracy and fidelity to the original conversations.

### Data Analysis

The qualitative data from the FGDs were analyzed using NVivo 12 (Lumivero) software. A thematic analysis approach was employed to identify patterns and themes within the data. The analysis followed the five stages of data analysis: familiarization, identifying a thematic framework, indexing, charting, and mapping and interpretation [38].

**Figure 1.**
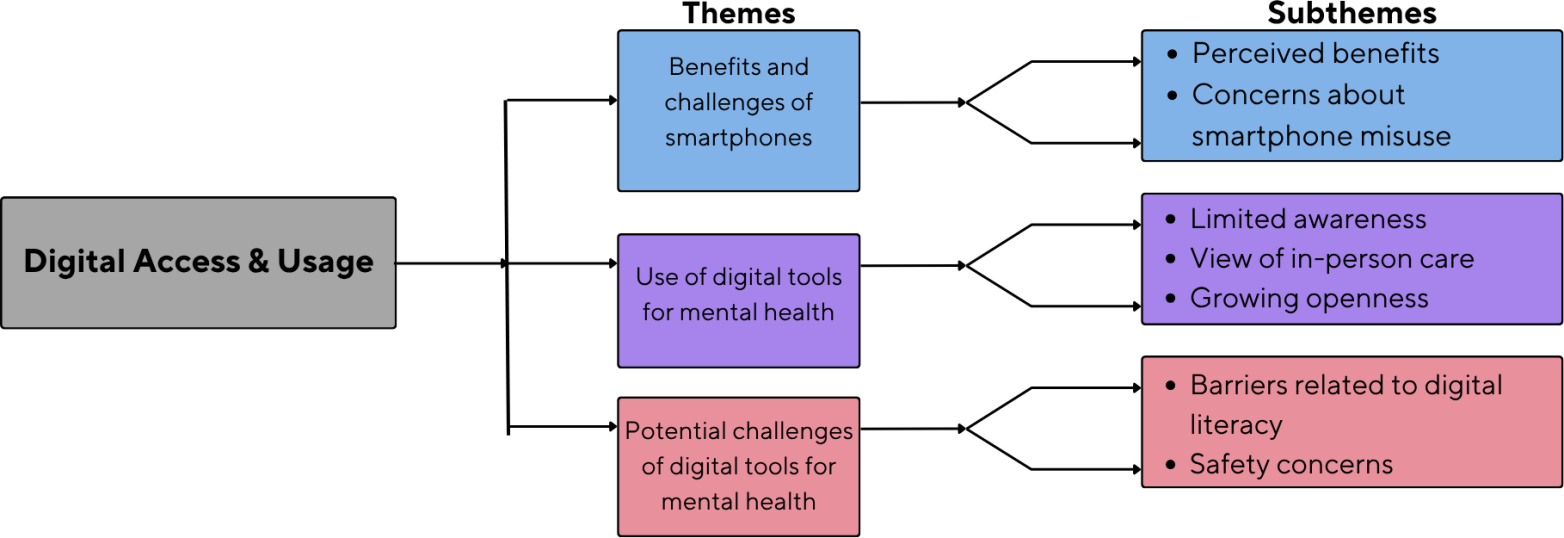
Coding tree illustrating key themes and subthemes derived from the thematic analysis. Focus group discussions on smartphone use and perceptions of digital mental health tools among individuals with serious mental disorders and their caregivers in Korail slum, Dhaka, Bangladesh.

Each transcript was read multiple times to enhance familiarity with the data. Initial thoughts, reflections, and preliminary codes were noted. The transcript was then read again, and preliminary themes were recorded. Preliminary themes were then grouped into clusters based on common features and meanings. A coding tree was developed to systematically organize the themes, capturing the hierarchical relationships between main themes, subthemes, and associated codes. This framework enabled a nuanced analysis of participants’ perspectives and experiences. The coding structure was iteratively refined through regular team discussions, and each theme was cross-checked against the transcripts to ensure accurate representation of the data. This process was applied consistently across all transcripts to enhance the rigor and coherence of the analysis. The final coding tree is presented in Figure 1. These themes were validated by cross-checking with the transcript to ensure they accurately represented the data. This process was repeated for each transcript to maintain consistency. The themes from all transcripts were then compared and combined into master themes to create a comprehensive portrayal of the participants’ experiences. The master themes were thoroughly reviewed against the transcripts to ensure they accurately reflected and were firmly rooted in the participants’ accounts. Commonalities among the preliminary themes were identified and represented as subthemes, reflecting lower-order aspects of the master themes. To enhance confirmability [39], the analysis was grounded in participants’ accounts through the use of direct, anonymized quotes that illustrate each theme. A clear audit trail was maintained throughout, documenting coding decisions, thematic development, and reflections in analytical memos to ensure dependability [39]. The final coding framework was reviewed and refined collaboratively by multiple researchers to ensure consistency and rigor, with any discrepancies resolved through discussion and consensus.

Reflexivity [40] was integrated throughout the analytical process. The research team actively reflected on their positionalities, including cultural backgrounds and professional roles, to remain aware of how these factors might shape data interpretation. Regular team discussions were held to critically assess assumptions and ensure the analysis remained anchored in participants’ lived experiences. To support credibility [39], emerging themes were continuously reviewed through peer debriefing and iterative comparisons across focus groups. This process helped validate thematic consistency and ensure findings captured the breadth of perspectives shared by both individuals with SMDs and their caregivers.

Transferability [39] was supported by providing rich contextual detail about the study setting and participant demographics. Descriptions of life in Korail slum, including social and infrastructural challenges, offer important context for assessing how these findings may resonate with other low-resource, urban communities in Bangladesh and similar LMIC settings [2,41]. The transparent analytical process and inclusion of participant narratives further support the relevance of these findings for broader application.

## Results

### Demographic Information

A total of 8 FGDs were conducted, each group comprising 4‐6 participants who were either people with lived experiences or caregivers. The total number of participants for the 8 FGDs was 38 (Table 1). The average age of participants was 37 (SD 13.7) years, mostly comprising female participants. Out of the 38 participants, 17 (45%) personally owned a smartphone, while 35 (92%) reported having access to one within their household. The average duration of personal smartphone ownership was 3.3 (SD 1.15) years, with most users relying on low-cost Android phones.

**Table 1.** Participant demographic information (N=38).

Characteristics	Count
Age (y), mean (SD)	37 (13.7)
Gender (female), n (%)	30 (79)
Education level, n (%)	
No education	5 (13)
Primary	11 (29)
Middle school	16 (42)
High school	4 (11)
Undergraduate	2 (5)
Marital status, n (%)	
Married	29 (76)
Unmarried	4 (11)
Widowed	4 (11)
Divorced	1 (2)
Employed, n (%)	
Yes	12 (32)
No	26 (68)
Smartphone ownership, n (%)	
Yes	17 (45)
No	21 (55)
Average time of Smartphone ownership (y), mean (SD)	3.33 (1.15)
Smartphone ownership in family, n (%)	
Yes	35 (92)
No	3 (8)

### Overview of Qualitative Themes

We found three major themes: smartphone use and benefits, digital awareness and attitudes, and challenges in using digital tools for mental health (Figure 1). These are expanded on further below.

### Benefits and Challenges of Digital Tools

#### Perceived Benefits

Overall, participants were familiar with digital tools for other parts of their lives and acknowledged its convenience within everyday life. For instance, one participant mentioned, “Before we had to stand at the bank all day to withdraw disability allowance now it directly comes to the phone.” Participants emphasized the importance of communication, video calls, and messaging apps in maintaining relationships, especially with family members living abroad, and the ability to access news and information through social media platforms like Facebook and WhatsApp was also highlighted as a significant benefit. One stated, “People before used to send letters to seek updates, it took time to hear back. Now it is easy to know about people (using smartphones).” Another participant added, “Communication with friends and family is much easier with smartphones.” There was also discussion about convenience of access to information using smartphones. One participant added, “Phones are used for accessing information about health and various services.” They expressed that access to timely and relevant information is valued and seen as potentially helpful, particularly when it is available in a convenient and familiar format.

#### Concerns About Smartphone Misuse

Participants highlighted various ways in which smartphones can be misused, particularly by adolescents. Concerns were raised about inappropriate content, the facilitation of risky behaviors, and negative impacts on academic performance. Others highlighted concerns about addiction, particularly among the younger generation, as children and teenagers spend excessive amounts of time on their phones, often at the expense of studying or socializing with family. Another participant expressed frustration with the excessive gaming and its impact: “Every kid is so busy playing games, it seems that they have no time to pick up the call from family,” and another added, “Whole day they (younger family members) are on it (phones), even if you slap them, they will not let it go.”

### Use of Digital Tools for Mental Health

#### Limited Awareness of Digital Mental Health Tools

Despite familiarity of digital tools, awareness of digital mental health tools was generally low among the participants. Many were unaware of the existence of apps and online services designed to provide mental health support. One participant stated simply, “I have no idea” when asked about health services through phones. Another echoed, “No, I don’t know” about apps for mental health. Another participant mentioned, “We directly communicate with the doctor; no service was ever taken over the phone.” This lack of knowledge extended to the potential benefits of such tools, with several participants indicating that they had never used or even heard of mental health apps, “No, I have never used any such app.” However, one participant shared, “I once spoke to an Indian doctor through an app. He told me how to talk to my sister-in-law, how to behave, all of this.”

#### View of In-Person Care

Despite the potential of digital tools, many participants preferred traditional methods of seeking mental health care. They commonly visited doctors in person and relied on face-to-face consultations for treatment and advice. One participant shared, “We go to a doctor; the doctor gives one month of medicine.” This preference was attributed to the trust and reassurance that comes with direct interaction with health care professionals.

#### Growing Openness to Mobile-Based Mental Health Support

While most participants had limited experience with online consultations, there was a general interest in exploring this option. One participant added, “Consulting a doctor through an app can be very helpful.” Some expressed the view that online consultations could save time and reduce the costs associated with traveling to health care facilities. One participant noted, “Online consultations could save us a lot of travel time and cost.” The convenience of receiving medical advice from home was highlighted, especially for those with mobility issues or residing in remote areas. The potential of health apps to provide immediate assistance and support was recognized by several participants. They suggested that apps could offer valuable information on managing mental health conditions, including treatment schedules and medication reminders, with one stating, “If counseling, doctor’s advice, what kind of treatment should be given to them can be provided (through phones), it would be good.” Participants also noted that health apps could serve as a resource for caregivers, providing guidance on how to care for individuals with mental health issues, “health apps can guide us on how to care for our loved ones better.” Another participant reflected on the need for such tools, saying, “It would be great if there is a service available through the app for such problems,” acknowledging the limitations they face in accessing in-person services. Caregivers also recognized the potential benefits of digital tools in guiding them on how to care for individuals with mental health issues: “Health apps can guide us on how to care for our loved ones better.” This indicates that digital tools can serve as valuable resources for those supporting individuals with mental health challenges. The general sentiment was positive toward the potential of these services, with one participant mentioning, “If I get advice from a good doctor from home, it will be good,” and another stating, “Consulting a doctor through an app can be very helpful.”

### Potential Challenges

#### Barriers Related to Digital Literacy and Usability

Despite widespread appetite for digital mental health tools, participants acknowledged challenges such as the digital divide and lack of technological literacy, and concerns about the reliability of online services were also mentioned. One participant highlighted, “It saves time and travel costs but not everyone knows how to use the apps properly.” Another added, the importance of addressing these challenges to ensure the successful implementation of digital mental health interventions was emphasized, “It will be good, but we need proper guidance on how to use these services.”

#### Safety Concerns for Individuals With Mental Illness

Participants also expressed concerns about the usability of these tools for older family members, stating, “It can be challenging to teach older family members how to use health apps.” The need for user education and support to maximize the effectiveness of these digital tools was stressed. Furthermore, participants discussed the risks for mentally ill individuals using digital tools. One participant shared an experience: “When my sister-in-law was sick and had the phone on her, she used to make such bad comments, leave bad images, and she used to make very bad posts on people’s profiles.” Another participant emphasized the potential risks: “Mentally ill patients can do many things on the phone without realizing it....Harassment can be due to political reasons.” These accounts highlight the importance of considering the specific vulnerabilities of certain user groups when developing and implementing digital health interventions. Ensuring that appropriate safeguards and support mechanisms are in place is crucial to mitigate these risks.

## Discussion

### Principal Results

This study explored the perceptions and awareness of digital mental health tools among residents of Korail slum in Dhaka, Bangladesh. Participants were generally unaware of existing mental health apps but expressed strong interest in using mobile-based tools if appropriate support, content, and guidance were available. Key barriers included low digital literacy, stigma, and a preference for face-to-face consultations, while convenience and cost-saving were cited as major facilitators.

The findings reveal a general lack of awareness and understanding of digital mental health tools among the residents of the Korail slum. Many participants were unaware of the existence of apps and online services designed to provide mental health support, which highlights a significant gap in mental health literacy within the community. Despite this lack of awareness, there was a noticeable interest in the potential of digital mental health tools, with participants acknowledging that these tools could provide timely and cost-effective support, reducing the need for frequent hospital visits and associated expenses.

Several barriers to the utilization of digital mental health tools were identified, including technological literacy and accessibility. The digital divide was a prominent concern, with participants highlighting the lack of technological literacy as a significant barrier. This issue is compounded by the challenges of teaching older family members how to use these tools. Additionally, concerns about the reliability and effectiveness of online services were mentioned, and traditional doctor visits are still preferred by many due to the trust and reassurance that comes with face-to-face interactions [42]. This preference may reflect deeper cultural and perceptual factors related to mental health [43,44] rather than a direct rejection of digital mental health interventions. In contexts where mental illness is often stigmatized, seeking face-to-face care from a trusted physician can offer a sense of legitimacy and reassurance [25,43,45]. Additionally, low awareness of mental health as a medical issue or tools to support this issue may lead to underappreciation of the value of remote or digital forms of care [44,45]. These findings suggest that efforts to introduce digital interventions should be accompanied by broader awareness and stigma reduction campaigns to reframe how mental health is understood and to position digital tools as complementary rather than substitutive to traditional care.

Despite these barriers, several facilitators to the utilization of digital mental health tools were identified. Participants recognized the potential of these tools to make health care more accessible, especially for those who cannot travel. This reflects the potential for digital tools to bridge the gap in mental health services, particularly in underserved areas like slums. The study also highlighted the importance of providing proper guidance and education on using these services. Participants stressed the need for user education and support to maximize the effectiveness of digital tools. This suggests that initiatives aimed at improving technological literacy and providing support could significantly enhance the adoption and effectiveness of digital mental health interventions [29].

Cultural beliefs, norms, and attitudes toward mental illness and technology can significantly influence the acceptability and use of digital mental health tools across LMICs [20,46,47]. While our study reflects the context of a Bangladeshi urban slum, where religious values, stigma, and informal care networks play a central role, these dynamics may differ in other regions [11]. For example, communities in sub-Saharan Africa may have different levels of mHealth adoption, while Latin American settings may feature stronger public mental health infrastructures or community health outreach programs. Such differences may shape trust in digital platforms, preferences for in-person versus remote care, and openness to app-based interventions. Therefore, further context-specific studies are needed to examine how cultural, technological, and health system factors affect digital mental health engagement across diverse LMICs.

While participants were introduced to both a telemedicine-style app (Doctime) and an autonomous, informational app (Maya Apa), most responses focused on the former. This suggests a preference or at least greater conceptual familiarity with real-time, doctor-driven models. The limited commentary on autonomous, non-telemedicine interventions likely reflects both unfamiliarity with such models and cultural expectations around personal interaction in health care. These perceptions are important, as they highlight a potential barrier to the implementation of scalable, self-guided digital mental health tools in this context. As the field moves toward autonomous solutions for scalability, especially in LMICs, our findings suggest that greater user education and co-design with target communities may be necessary to improve acceptability and uptake of these newer models of care.

These findings add to the growing body of evidence on the acceptability of digital mental health tools in low-resource settings. While global momentum is increasing around mHealth innovations, this study highlights the need for contextualized, culturally appropriate, and socially supported implementation strategies in urban slum environments.

### Ethical Considerations in Implementations

Implementing digital mental health tools in low-resource settings presents several ethical challenges that must be carefully addressed to ensure safe and equitable access. As participants in this study highlighted, concerns about privacy, misuse of technology, and safety, particularly for individuals with SMDs, can pose real risks. In contexts where digital literacy is limited and access to trusted online health information is inconsistent, users may misinterpret content, misuse apps, or fall victim to misinformation or exploitation. These risks are amplified in environments with weak data protection infrastructure or low public awareness of digital rights [25]. Moreover, reliance on technology without adequate support mechanisms may marginalize users with low literacy, older adults, or individuals with cognitive impairments [48,49]. To mitigate these risks, digital mental health tools should be developed with clear privacy safeguards, culturally appropriate guidance, and integrated referral pathways for offline support. Involving local stakeholders and end-users in co-design is critical to ensure ethical alignment and social acceptability.

### Limitations

Despite the valuable insights provided by this study, several limitations need to be acknowledged. First, the study was conducted in a single slum community in Dhaka, Bangladesh, which may limit the generalizability of the findings to other slum areas or LMICs with different sociocultural contexts [50]. The sample size, though appropriate for qualitative thematic analysis, is relatively small (n=38), which may limit the breadth and variability of perspectives captured [50]. Second, the study relied on self-reported data from FGDs, which may introduce biases such as social desirability bias, where participants might overstate positive aspects or underreport negative aspects to conform to perceived expectations [29]. Furthermore, while purposive sampling was appropriate for the exploratory and context-specific aims of this study, we acknowledge that it may have introduced some degree of selection bias. Participants were recruited through trusted community representatives affiliated with the TRANSFORM project [11], which likely facilitated openness and trust during data collection. However, this approach may have favored individuals who were more socially engaged or willing to discuss mental health–related topics in a group setting. As with many qualitative studies in marginalized communities, those with the most severe symptoms or highest levels of stigma may have been less likely to participate [51]. While the sample captured a diverse range of perspectives, future research could incorporate additional recruitment strategies such as household outreach or individual interviews to ensure broader inclusion. While our findings offer valuable insights into community-wide perceptions of digital mental health tools, we were unable to explore differences between smartphone owners and nonowners. Although we collected demographic data on smartphone access, transcripts from the FGDs were anonymized and not linked to individual participants’ ownership status. As a result, it was not feasible to systematically compare viewpoints across these two groups. Given that personal experience with smartphones may significantly shape perceptions of digital health interventions, future research using individually linked or interview-based data could help unpack these differences. This would allow for a more nuanced understanding of how digital familiarity influences the acceptability and perceived utility of mobile mental health tools in slum settings.

Additionally, the lack of awareness and understanding of digital mental health tools among participants could have influenced their responses, potentially limiting the depth of insights regarding the barriers and facilitators to utilizing these tools [33]. Technological literacy was identified as a significant barrier, yet the study did not quantitatively assess the participants’ actual proficiency with digital tools. This gap indicates a need for future research to include objective measures of technological literacy to better understand this barrier [19]. As this was a focus group study designed to encourage open dialogue within groups rather than attribute views to individual characteristics, we did not analyze responses based on smartphone ownership status. While this approach captured a collective understanding of digital mental health perceptions in the community, future studies may benefit from comparing views across subgroups, such as smartphone users and nonusers, to better tailor digital interventions. Additionally, while full data saturation may not have been achieved, several key themes, such as attitudes toward smartphone use, digital literacy challenges, and preferences for traditional care, emerged consistently across the FGDs. The final two discussions reinforced earlier findings, suggesting thematic convergence. Given the formative and exploratory nature of this study, the insights remain valuable and provide a foundation for future qualitative work. This study was conducted in a single urban slum in Dhaka, Bangladesh, which may limit the extent to which the findings can be generalized to other LMIC contexts. However, many of the challenges identified, such as stigma, low mental health literacy, and limited digital access, are common across underserved urban settings in other LMICs [2,4,11,25,45]. As such, the insights gained may still be relevant and transferable. Future research should aim to include multiple communities across different regions or countries to better capture contextual variation and enhance generalizability. While this study provides foundational insights into the awareness and perceptions of digital mental health tools in a slum setting, its limitations highlight the need for further research to expand these findings and address the identified gaps.

### Comparison to Prior Work

This study adds to a growing body of literature demonstrating the promise of digital mental health interventions in resource-constrained settings, particularly within LMICs. Our findings are consistent with previous work showing that digital tools can expand access, reduce treatment gaps, and provide cost-effective support in communities with limited formal mental health infrastructure [23,25,46,47,52] However, our study extends this literature by focusing on an underserved slum community where the digital health ecosystem remains nascent. In contrast to digital health successes reported in countries where mental health apps and mobile services have shown promising engagement [27,53,54], our participants demonstrated limited awareness of digital mental health tools and low technological literacy. For instance, the Enhanced mHealth mental health screening for adolescents living with HIV transitioning into adult care (EMMATAC) study conducted in Nairobi, Kenya, assessed a self-administered mHealth screening tool among adolescents and young adults living with HIV [54]. The study reported high feasibility (83%) and acceptability (80%), with a significant prevalence of mental health conditions such as post-traumatic stress disorder and depression among participants [54]. Similarly, the A Sequential, Multiple Assignment Randomized Trial (SMART) for Non-Specialist Treatment of Common Mental Disorders in Kenya: Leveraging the Depression and Primary-Care Partnership for Effectiveness-Implementation Research (DAPPER) Project (SMART-DAPPER) in western Kenya evaluated the delivery of psychotherapy and medication for depression and post-traumatic stress disorder via mHealth [27]. Several participants preferred mHealth over in-person treatment, citing affordability and convenience as primary reasons [27]. These findings suggest that while digital interventions may be viable at a systems level, community-level barriers persist that hinder their equitable adoption and meaningful use.

Our findings align with broader evidence from other LMICs. For example, previous studies demonstrated the effectiveness of mHealth interventions for improving mental health outcomes in rural Indian communities, particularly when combined with community-based education and engagement strategies [28]. However, concerns about the usability, reliability, and safety of digital platforms, particularly for individuals experiencing SMDs, were consistent with previously reported challenges in implementing digital mental health intervention [21]. This highlights the importance of designing interventions that are not only technologically accessible but also culturally resonant and ethically secure. Additionally, our study supports previous findings that the successful adoption of digital mental health interventions in marginalized communities requires more than mere access to technology [34,49]; it also demands a nuanced understanding of social contexts, trust dynamics, and user capabilities. Without deliberate efforts to address these factors, digital tools risk perpetuating rather than reducing existing health inequities.

In summary, while our findings are consistent with global evidence that digital tools hold promise for expanding mental health care in LMICs, they also highlight the importance of grounding digital health interventions in the lived realities of the communities they aim to serve. Tailored educational initiatives, community involvement in design and delivery, and safeguards for vulnerable users are essential to ensure these tools are not only available but also accessible, acceptable, and effective in slum settings.

### Conclusion

Based on our findings, we propose several practical recommendations for developing and implementing digital mental health tools in low-resource slum settings. First, interventions should be accompanied by structured digital literacy training, particularly targeting older adults and individuals with limited experience using mobile technology. Second, culturally relevant content in local languages is critical to improve usability and engagement. Third, involving caregivers and community members in the design and delivery process can build trust and ensure that tools align with community needs. Fourth, mental health apps should include safeguards to support users with SMDs, such as guidance for caregivers and emergency contact features. Finally, these tools should be positioned as supplements to rather than replacements for face-to-face care to increase acceptance among users who strongly value in-person consultations.

This study shows people with lived experience are receptive and interested in the transformative power of digital tools for mental health care in slum settings like Korail. However, for these tools to be effective, it is crucial to address the existing barriers and provide adequate support and education to users. By doing so, digital interventions can become a significant solution to the mental health care challenges faced by underserved communities in LMICs. The findings highlight the need for further research and investment in digital mental health solutions tailored to the unique needs and contexts of slum populations.

## Abbreviations

EMMATAC: Enhanced mHealth mental health screening for adolescents living with HIV transitioning into adult care
FGD: focus group discussion
LMIC: low- and middle-income country
mHealth: mobile health
SMART-DAPPER: A Sequential, Multiple Assignment Randomized Trial (SMART) for Non-Specialist Treatment of Common Mental Disorders in Kenya: Leveraging the Depression and Primary-Care Partnership for Effectiveness-Implementation Research (DAPPER) Project
SMD: serious mental disorder
TRANSFORM: Transforming Access to Care for Serious Mental Disorders in Slums
